# NMDA Receptor Hypofunction Leads to Generalized and Persistent Aberrant γ Oscillations Independent of Hyperlocomotion and the State of Consciousness

**DOI:** 10.1371/journal.pone.0006755

**Published:** 2009-08-25

**Authors:** Tahir Hakami, Nigel C. Jones, Elena A. Tolmacheva, Julien Gaudias, Joseph Chaumont, Michael Salzberg, Terence J. O'Brien, Didier Pinault

**Affiliations:** 1 Department of Medicine, Royal Melbourne Hospital, University of Melbourne, Parkville, Australia; 2 INSERM U666, Physiopathologie et psychopathologie cognitive de la schizophrénie, Université de Strasbourg, Faculté de Médecine, Strasbourg, France; 3 Department of Psychiatry, St Vincent's Hospital, University of Melbourne, Fitzroy, Victoria, Australia; Chiba University Center for Forensic Mental Health, Japan

## Abstract

**Background:**

The psychotomimetics ketamine and MK-801, non-competitive NMDA receptor (NMDAr) antagonists, induce cognitive impairment and aggravate schizophrenia symptoms. In conscious rats, they produce an abnormal behavior associated with a peculiar brain state characterized by increased synchronization in ongoing γ (30–80 Hz) oscillations in the frontoparietal (sensorimotor) electrocorticogram (ECoG). This study investigated whether NMDAr antagonists-induced aberrant γ oscillations are correlated with locomotion and dependent on hyperlocomotion-related sensorimotor processing. This also implied to explore the contribution of intracortical and subcortical networks in the generation of these pathophysiological ECoG γ oscillations.

**Methodology/Principal Findings:**

Quantitative locomotion data collected with a computer-assisted video tracking system in combination with ECoG revealed that ketamine and MK-801 induce highly correlated hyperlocomotion and aberrant γ oscillations. This abnormal γ hyperactivity was recorded over the frontal, parietal and occipital cortices. ECoG conducted under diverse consciousness states (with diverse anesthetics) revealed that NMDAr antagonists dramatically increase the power of basal γ oscillations. Paired ECoG and intracortical local field potential recordings showed that the ECoG mainly reflects γ oscillations recorded in underlying intracortical networks. In addition, multisite recordings revealed that NMDAr antagonists dramatically enhance the amount of ongoing γ oscillations in multiple cortical and subcortical structures, including the prefrontal cortex, accumbens, amygdala, basalis, hippocampus, striatum and thalamus.

**Conclusions/Significance:**

NMDAr antagonists acutely produces, in the rodent CNS, generalized aberrant γ oscillations, which are not dependent on hyperlocomotion-related brain state or conscious sensorimotor processing. These findings suggest that NMDAr hypofunction-related generalized γ hypersynchronies represent an aberrant diffuse network noise, a potential electrophysiological correlate of a psychotic-like state. Such generalized noise might cause dysfunction of brain operations, including the impairments in cognition and sensorimotor integration seen in schizophrenia.

## Introduction

The symptoms of schizophrenia are underlain by neuronal mechanisms that are poorly understood. It is currently thought that they result, to some extent, from functional disconnections in cortical-related networks, which denote the disintegration of psychic processes [1]. Several hypotheses regarding the underlying pathophysiological mechanisms have been proposed [2], [3]. Growing evidence for hypofunction of N-methyl d-aspartate-type glutamate receptors (NMDAr) in schizophrenia has been accumulating [4]–[7]. Consistent with this, a single non-anesthetic dose of non-competitive NMDAr antagonists, such as ketamine and phencyclidine, can induce psychotic symptoms (including hallucinations) and cognitive abnormalities reminiscent of those seen in schizophrenia and exacerbate symptoms in schizophrenic patients [8]–[11]. The neuronal mechanisms underlying hypofunction of NMDAr, and how these are related to the psychotic symptomatology, remain to be determined. In the conscious rat, a single non-anesthetic injection of ketamine or MK-801 significantly increases the power and intrinsic frequency of wake-related, spontaneously occurring, cortical γ frequency (30–80 Hz) oscillations [12]. The NMDAr hypofunction-related pathophysiological cortical γ oscillations are accompanied by abnormal behavior, including hyperlocomotion and ataxia. These may correspond to some of the motor abnormalities observed in neuroleptic naïve schizophrenic patients, although the latter tend to be much more subtle [13]–[16]. Therefore, the aim of the present study was to determine whether or not ketamine-induced aberrant cortical γ oscillations were 1) correlated with quantitative measures of locomotion and 2) caused by conscious or unconscious premotor/sensorimotor neuronal activity related to hyperlocomotion. Answering these important questions allows the hypothesis that “NMDAr hypofunction-induced hyperlocomotion and/or aberrant ongoing γ oscillations are associated to a psychotic-like state” to be tested. The first question was addressed by combining, in freely moving rats, electrocorticographic (ECoG) recording and computer-assisted video tracking to quantify simultaneously the motor and ECoG changes in response to the administration of a single non-anesthetic low dose of ketamine or MK-801, the latter molecule being a more specific non-competitive NMDAr antagonist than the former one. The second question was addressed by assessing, using multiple recordings, the psychotomimetic action of these NMDAr antagonists on spontaneously occurring γ oscillations in cortical and subcortical structures in diverse consciousness states produced by sedative and anesthetic substances.

Another central issue was to relate the natural and NMDAr antagonist-induced aberrant γ oscillations recorded with surface ECoG electrodes to the current sources or generators. Because of volume conduction and network properties, we assume that the cortical electrodes recorded integrated population activities, directly from multiple cortical generators and, directly and indirectly (e.g., via thalamocortical neurons), from subcortical generators [17]. So, the possible contribution of intracortical and subcortical networks in the recorded surface ECoG was addressed using multisite recordings.

## Results

### 1. Ketamine and MK-801 induce temporally correlated hyperlocomotion and aberrant γ oscillations

**Figure 1:**
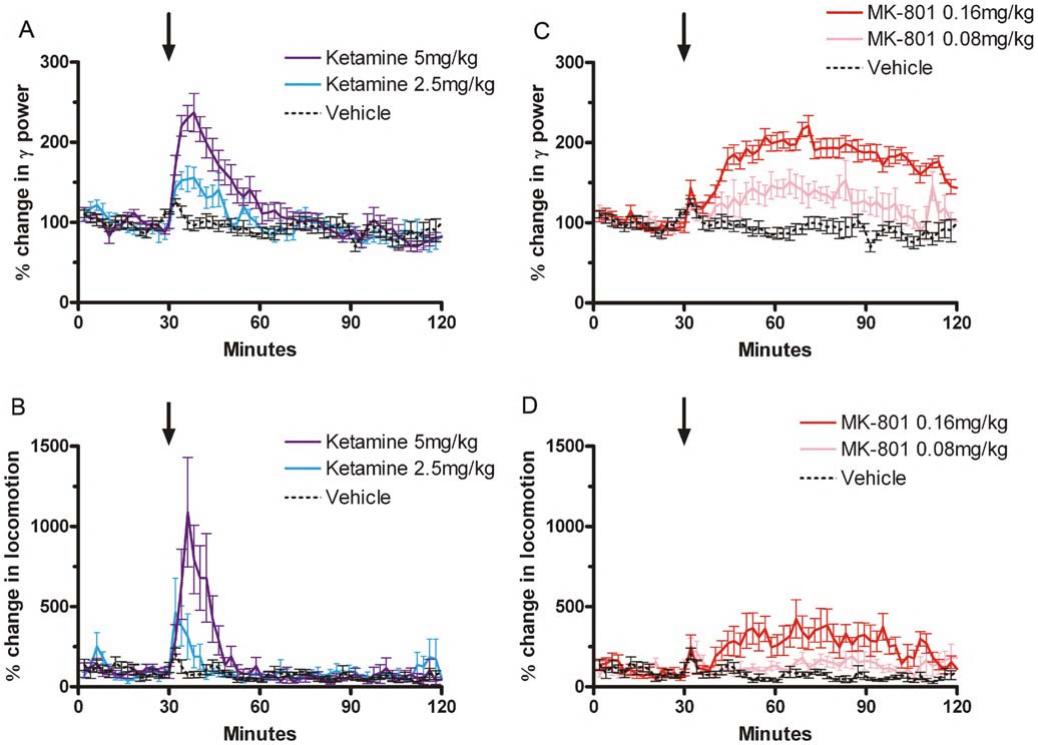
Ketamine and MK-801 induce parallel and dose-dependent increases in γ (30–80 Hz) power (A and C) and locomotor activity (B and D) in freely moving rats. Ketamine (2.5 and 5 mg/kg) or MK-801 (0.08 and 0.16 mg/kg) were injected subcutaneously. Black arrows indicate the injection time. Data are presented in 2-minute intervals, and represent the mean (±s.e.m.) percentage response compared to the 30 minute habituation period. Gamma power measurements represent the means of those obtained from the left and right hemispheres.

The current experiments were conducted in freely moving rats to study the degree of correlation of changes in γ power and locomotion in conscious rats treated with a single non-anesthetic dose of ketamine or MK-801 (Fig. 1). Administration of ketamine produced a significant dose-dependent and immediate increase in both γ power and locomotor activity (Fig. 1A–B), which persisted for 30 minutes before returning to baseline levels. The peak γ power response occurred 8 minutes after injection, and was significantly increased compared to control levels at this time (*F*_(2, 24)_ = 24.56, *p*<0.0001), while the peak locomotor response occurred slightly earlier at 6 minutes after injection and was also significantly higher than vehicle-treated rats at this point (*F*_(2, 24)_ = 5.981, *p* = 0.0133). Furthermore, injection of ketamine produced a significant increase in total response as measured via the AUC when assessing γ power (*F*_(2, 24)_ = 18.91, *p* = 0.0001) and locomotor activity (*F*_(2, 24)_ = 8.625, *p* = 0.0036), during the drug-active period. From visual observations, the ketamine-treated rats had consistent short-term hyperactive behavior (running, crawling, and irritability) and ataxic-like behavior (unsteady gait, rearing then falling down on the back).

Administration of MK-801 also produced a significant and dose-dependent increase in both γ power and locomotor activity (Fig. 1C–D), which followed a different time course to that induced by ketamine. The drug response was initiated 10 minutes following administration, and persisted for the duration of the recording period (90 minutes post-injection). The peak γ power response occurred 41 minutes after injection and was significantly increased compared to control levels at this time (*F*_(2, 24)_ = 39.27, *p*<0.0001), while the peak locomotor response occurred slightly later at 46 minutes following injection, and was also significantly higher than control (*F*_(2, 24)_ = 10.36, *p* = 0.0017). Furthermore, injection of MK-801 produced a significant increase in total response as measured via the AUC when assessing γ power (*F*_(2, 24)_ = 47.68, *p*<0.0001) and locomotor activity (*F*_(2, 24)_ = 14.82, *p* = 0.0003), during the drug-active period. From visual observations, the MK-801-treated rats had mainly long standing hyperactive running type behavior.

**Figure 2:**
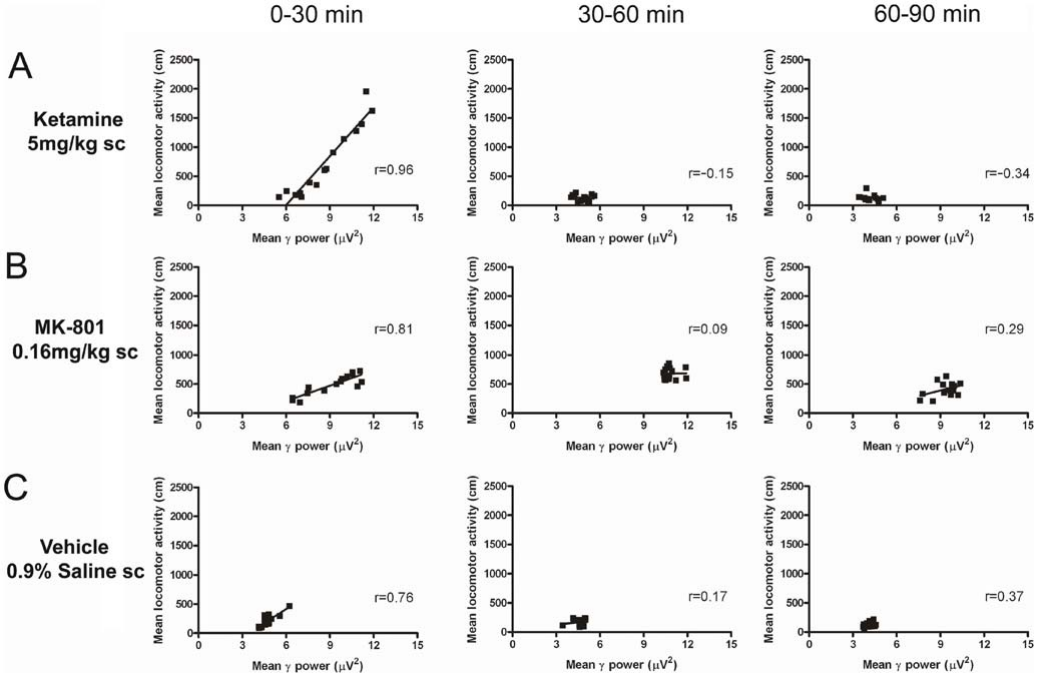
Group mean correlations comparing locomotor activity and γ power following administration of ketamine 5 mg/kg sc (A), MK-801 0.16 mg/kg sc (B), and vehicle (saline sc; C) in freely moving rats. Data are depicted in three 30-minute time windows (running left to right), and correlation coefficients calculated for these time frames for each drug. N = 8 for each group.

The clear temporal correlations linking γ power and locomotor activity following drug administration are depicted in Figure 2, with the strongest correlation (r = 0.96) observed between 0–30 minutes following ketamine (5 mg/kg) administration. Statistically significant differences were observed at this time point between the mean correlation coefficients obtained for the different treatments (*F*_(2, 8)_ = 6.43, *p* = 0.015), and post hoc analysis revealed this to be evident when comparing ketamine treatment to vehicle (Fig. 2A, left panel). At no other time point was significance observed when comparing the mean correlation coefficients (p>0.05).

### 2. Ketamine produces generalized ongoing γ hyperactivity over the surface of the cerebral cortex

**Figure 3:**
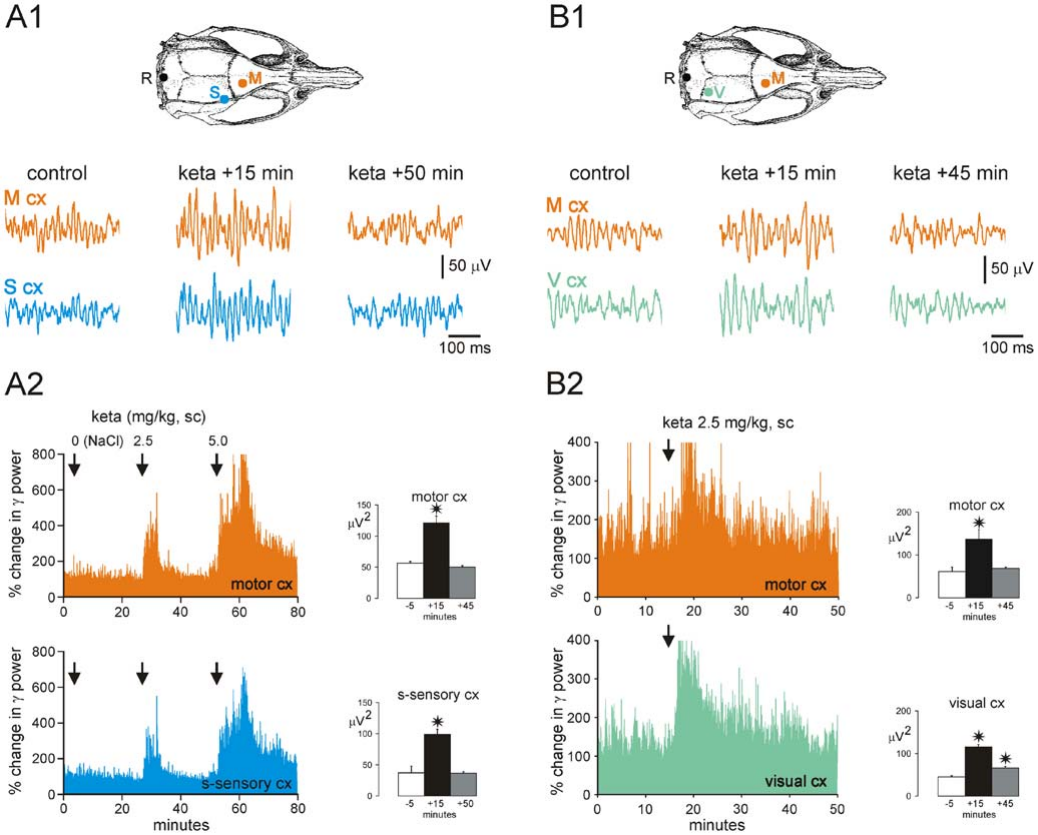
Ketamine increases the γ (30–80 Hz) power in the motor (M), somatosensory (S) and visual (V) cortices in free-moving rats. A1-2 and B1-2 are from two experiments. (A1 and B1) 500-ms episodes of paired M–S and M–V ECoG, respectively, containing γ bouts under control conditions (vehicle), 15 min and >45 min after subcutaneous (sc) injection of ketamine (2.5 mg/kg). The dorsal view of the cranium shows the location of the recording electrodes (R, reference). (A2 and B2) Simultaneous changes in γ power in the M and S cortices (A2) and in the M and V cortices (B2) before and after ketamine injection. The histograms (means±s.e.m.) show a significant γ increase in the M, S and V cortices about 15 minutes after ketamine administration, with full and partial recoveries ∼45 minutes later (comparison with the control condition; t-test with asterisk for p<0.001).

In an attempt to determine whether or not NMDAr-related γ hyperactivities are related only to the frontoparietal (sensorimotor) cortex, paired ECoG recordings were performed over either the frontal (motor) and parietal (somatosensory), or the frontal and occipital (visual) cortices (Fig. 3A1, B1). A single subcutaneous injection of ketamine (2.5 or 5.0 mg/kg) significantly increased the power of γ oscillations all over the recorded cortical areas (Fig. 3A2,B2; N = 6 rats).

### 3. Ketamine- or MK-801-induced γ hyperactivity is not caused by conscious sensorimotor processing underlying ataxic behavior and hyperlocomotion

**Figure 4:**
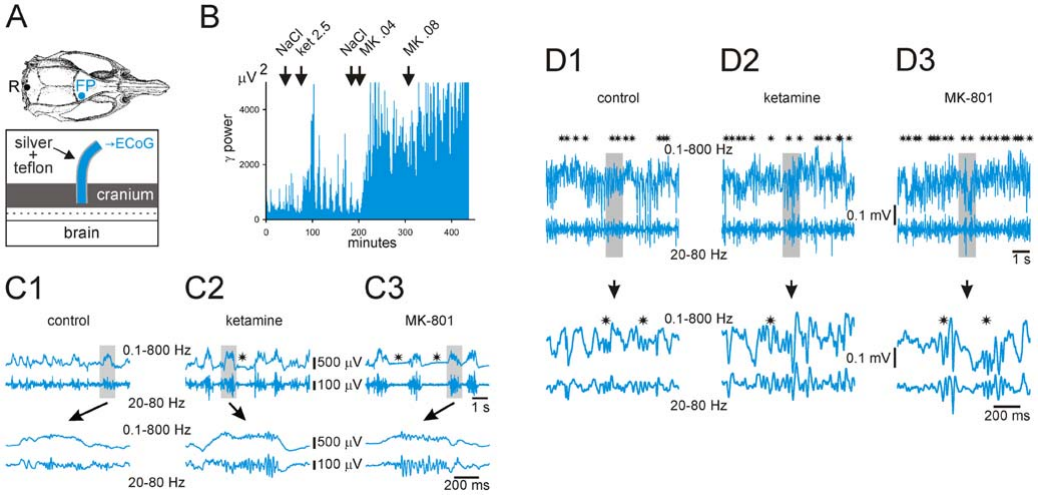
Ketamine or MK-801 increases both the power and the duration of ongoing ECoG γ oscillations under deep anesthesia. (A): Experimental design of the differential monopolar ECoG recording of the frontoparietal (FP) cortex relative to the reference (R) electrode, which is set to ground. The section of the silver wires (insulated with teflon) is put into the cranium (no contact with the meninges), whose contact is made easier with conductive paste. (B): Changes in γ power during a typical 8-hour experiment under urethane-anesthesia, during which the vehicle (NaCl, 0.9%, 1 ml/kg), ketamine (ket, 2.5 mg/kg) and MK-801 (MK, 0.04 and 0.08 mg/kg) were subcutaneously (sc) injected successively at different time (arrows). Note that this chart shows all the power values (resolution: 1.6 sec) of γ oscillations, occurring during and in between the slow waves. (C1–C3, top row): 8-s episode of the frontoparietal ECoG under urethane-anesthesia and three successive conditions (after injection of vehicle [NaCl, 0.9%], ketamine [7 min after injection of 2.5 mg/kg, sc] and MK-801 [18 min after injection of 0.08 mg/kg, sc]). (C1–C3, bottom row): the corresponding 1-s episodes (indicated by the grey back) showing the positive slow waves. The ECoG was recorded with two bandpasses (0.1–800 Hz and 20–80 Hz). The asterisks in (C2) and (C3) indicate periods of “electrical silence”. (D1–D3): A typical experiment done under deep pentobarbital anesthesia. Top row: 8-s episode of the frontoparietal ECoG under three conditions (after subcutaneous injection of vehicle [control], ketamine [20 min after injection of 5 mg/kg, sc] and MK-801 [40 min after 0.08 mg/kg, sc]). Bottom row: the corresponding 1-s episodes (indicated by the grey back). The ECoG was recorded with two bandpasses (0.1–800 Hz and 20–80 Hz). Asterisks indicate γ bursts.

Ketamine and MK-801 led to an abnormal brain state characterized by an increase in the power of baseline γ oscillations accompanied by abnormal motor activity, including hyperlocomotion and ataxia. Therefore, it was important to know whether this pharmacologically-induced γ hyperactivity was the consequence of conscious or unconscious sensorimotor processing underlying abnormal motor behavior. The effects of non-competitive NMDAr antagonists on neocortical γ oscillations were assessed in rats under four conditions that profoundly modified the state of consciousness. In deeply urethane-anesthetized rats, the sensorimotor ECoG (Fig. 4A) was characterized by low-frequency (0.77±0.06 Hz), medium- to high-voltage (0.2–1.0 mV), monophasic positive waves of variable duration (at the base: 439.29±54.86 ms; see Fig. 4C1). The wave's top had a variable morphology: one to a few peaks, a dome-like or a plateau-like waveform. It was often crowned with a long-lasting (302.64±22.30 ms) burst of γ oscillations, which were more persistent when compared to γ bouts recorded in freely moving rats (Table 1 and Fig. S1). A single non-anesthetic dose of ketamine or MK-801 significantly increased the γ power in such bursts (Fig. 4C1–C3 and Table 2), increased by a factor of 2–3 their duration (872.50±59.94 ms; t-test, p<0.0001) and slowed down the frequency of occurrence of the positive waves (after MK-801: 0.47±0.05 Hz; t-test, p = 0.0008). Furthermore, these NMDAr antagonists increased the frequency and duration of periods of apparent “electrical silence” that occasionally occurred in between positive waves (asterisks in Fig. 4C2,C3). It is worth noting that previous studies have reported that the power of γ oscillations recorded under anesthesia is significantly greater than that measured in the awaked basal state [18].

**Table 1:** Properties (means±s.e.m., N>30) of spontaneously occurring γ oscillations under different experimental conditions.

ECoG condition (see Fig. S1)	FREE	SEDATION	URETHANE	PENTO
duration (ms)	100.08±4.06	146.17±7.09	302.64±22.30	87.26±10.30
amplitude (µV)	44.42±1.34	97.83±3.21	78.06±6.68	116.11±0.01
γ power (µV^2^/Hz)	52.81±0.59	157.55±2.67	99.73±9.91	237.00±13.80
FAMP (Hz)	45.54±0.77	35.05±0.52	41.01±1.16	32.63±0.44

The values for the freely moving condition are from a previous study (Pinault, 2008). ECoG, electrocorticogram; FAMP, frequency at maximal γ power. FREE, drug-free awaked rats; SEDATION, fentanyl-haldol neuroleptanalgesia; URETHANE, urethane anesthesia; PENTO, pentobarbital-fentanyl anesthesia. Regarding the experimental conditions, details are available in Methods and in Fig. S1.

**Table 2:** Properties (means±s.e.m.; N>60, 2 rats; Student's t-test) of spontaneously occurring γ oscillations during slow waves under urethane-anesthesia after a single subcutaneous (sc) injection of saline, ketamine (∼10 min after injection) or MK-801 (∼20 min after injection).

	URETHANE NaCl (0.9%, 1 ml/kg, sc)	URETHANE ketamine (2.5 mg/kg, sc)	URETHANE MK-801 (0.08 mg/kg, sc)
γ power (µV^2^)	99.73±9.91	1275.50±81.61 P<0.0001	1445.11±73.00 P<0.0001
FAMP (Hz)	41.01±1.16	45.11±0.46 P<0.0001	38.69±0.55 P<0.0001

FAMP, frequency at maximal γ power.

Under deep pentobarbital anesthesia, the ECoG of the sensorimotor cortex was mainly characterized by complex sequences of slow (<15 Hz)/high-amplitude (>0.4 mV) waves, faster (>15 Hz)/low-amplitude (<0.2 mV) waves and of spike components (Fig. 4D1–D3). Among the faster waves, short-lasting (87.26±10.30 ms; Table 1 and Fig. S1) γ bouts were detectable and their amplitude was significantly increased following ketamine or MK-801 injection (Fig. 4D1–D3). The persistent character of background γ oscillations induced by NMDAr antagonists in freely moving rats could not consistently be reproduced under pentobarbital anesthesia. An apparent inverse dose-effect of pentobarbital on the amount of γ oscillations was observed (not shown).

**Figure 5:**
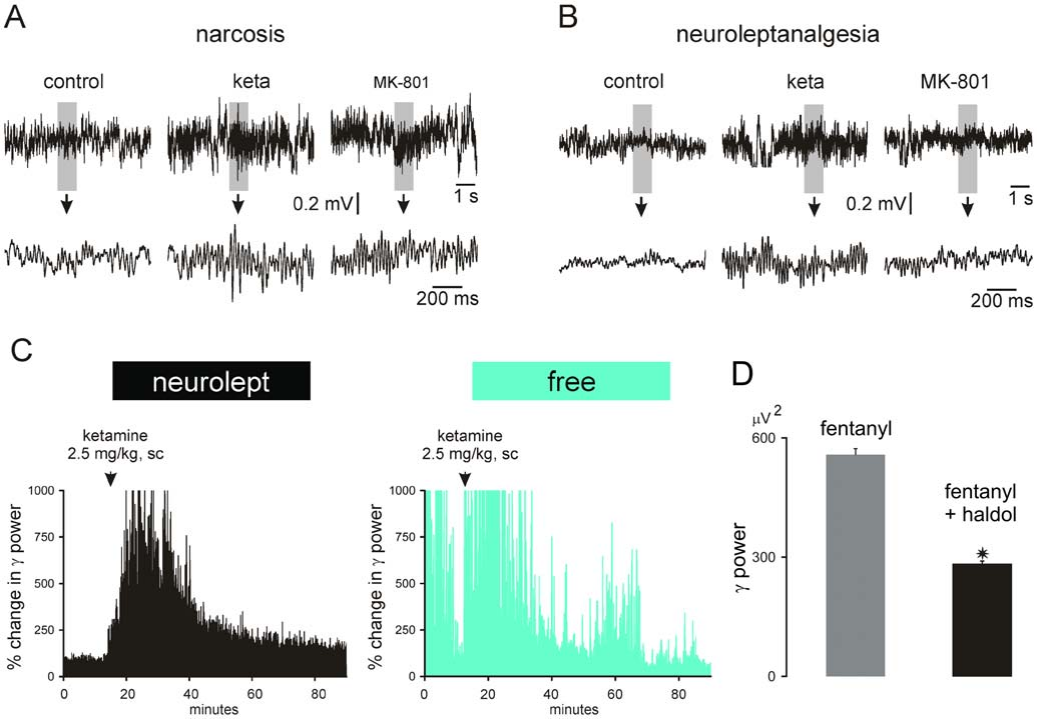
Ketamine or MK-801 increases the power of spontaneously occurring γ oscillations under sedation. The two NMDAr antagonists were tested under two sedative states, under fentanyl narcosis (A) or fentanyl-haldol neuroleptanalgesia (B). (A or B): 8-s episodes of the frontoparietal ECoG (bandpass: 0.1–800 Hz) under three conditions (after subcutaneous (sc) injection of vehicle [NaCl 0.9%; control], ketamine [∼15 min after injection of 2.5 mg/kg, sc] and MK-801 [∼40 min after injection of 0.08 mg/kg, sc]). The desynchronized 1-sec bout indicated by a grey back is expanded below (C): The charts represent % changes in γ power during a full recording session in a neuroleptanalgesied rat (left) and in a free-moving rat (right), which received a subcutaneous injection of ketamine. The chart of the free moving rat is contaminated by motion-induced cable artifacts, especially at the beginning of the recording session. (D): Amount of basal γ oscillations under narcosis (fentanyl; N = 250) and neuroleptanalgesia (fentanyl+haldol; N = 250). T-test with asterisk for p<0.0001.

The neurophysiological action of ketamine and MK-801 was also tested under two sedative states, under fentanyl narcosis [19] or fentanyl-haldol neuroleptanalgesia [19], [20]. The ECoG of the sensorimotor cortex usually alternated between medium-voltage slow oscillations (<0.5 mV, <15 Hz) and small-voltage faster oscillations (<0.2 mV, >15 Hz), but slow and fast oscillations could coexist. Under narcosis slow waves tended to be more persistent than under neuroleptanalgesia (not shown). During desynchronized states, the most prominent fast oscillations were bursts of rhythmic γ waves. They occurred at 0.2–3 Hz and were at least 2–3 times more powerful than those recorded in freely moving rats (Table 1 and Fig. S1). The intrinsic frequency in the γ bouts was significantly lower by ∼10 Hz than that measured in freely moving rats. Under sedation, ketamine or MK-801 increased the amount of cortical γ oscillations, at least during the desynchronized states (Fig. 5A,B), and slightly increased by ∼5 Hz on average the frequency at maximal γ power (Table 3). Under such experimental conditions, the NMDAr antagonist effects were dose-dependent and qualitatively similar whatever the route of injection (Fig. S2). The time course of the ketamine's or MK-801's effect was approximately comparable to the effect observed in freely moving conscious rats (Fig. 5C). Interestingly, the amount of basal γ waves was significantly lower under neuroleptanalgesia (fentanyl+haloperidol: 100-500 µV^2^/Hz) than under narcosis (only fentanyl: 200–800 µV^2^/Hz) (Fig. 5A,B and D). However, under our acute experimental conditions, the typical neuroleptic (300 µg/kg/h) did not prevent the psychotomimetic action of ketamine and MK-801 on the background γ oscillations (Fig. 5C).

**Table 3:** Properties (means±s.e.m.; N>80, 4 rats; Student's t-test) of spontaneously occurring γ oscillations during desynchronized states under fentanyl-haldol neuroleptanalgesia (SEDATION) after a single subcutaneous (sc) injection of saline (∼15 min), ketamine (∼15 min) or MK-801 (∼30 min).

	SEDATION NaCl (0.9%, 1 ml/kg, sc)	SEDATION ketamine (2.5 mg/kg, sc)	SEDATION MK-801 (0.08 mg/kg, sc)
γ power (µV^2^)	157.55±2.67	417.51±3.11 P<0.0001	491.25±4.18 P<0.0001
FAMP (Hz)	35.05±0.52	41.48±1.83 P<0.0001	39.27±1.03 P<0.0001

FAMP, frequency at maximal γ power.

### 4. Ketamine and MK-801 produce generalized and persistent aberrant γ oscillations in cortical and subcortical networks

**Figure 6:**
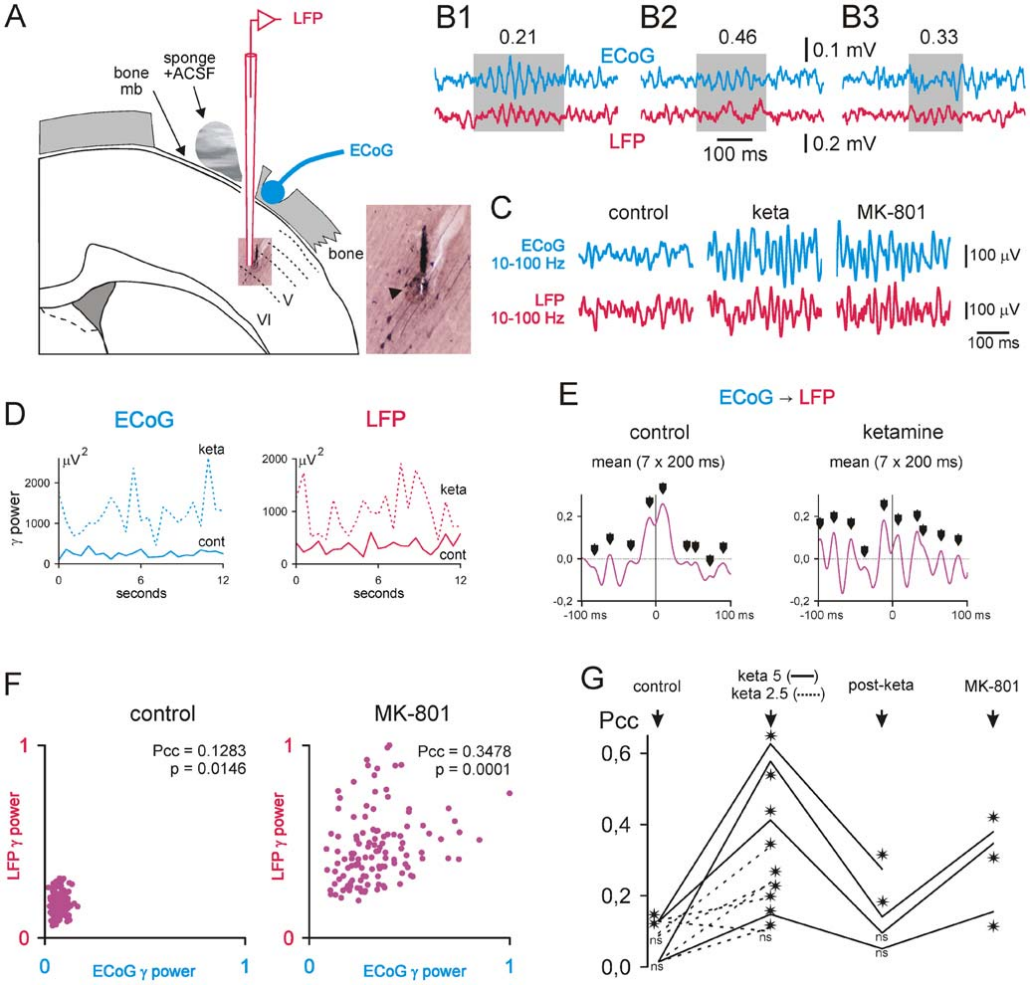
Ketamine or MK-801 increases the γ power in intracortical networks. These paired recordings were made under neuroleptanalgesia. (A): Experimental design showing simultaneous recordings of the extracellular local field potential (LFP) in the depth (layer V and VI) of the frontoparietal (or sensorimotor) cortex and of the related surface ECoG in sedated rats. The LFP is recorded with a glass micropipette (tip diameter: 7–15 um) containing ACSF and Neurobiotin (1%). At the end of the recording session, the neuronal tracer is applied using extracellular iontophoresis (+600 nA, 200 ms on, 200 ms off, for 10 min). The tracer is revealed using a standard ABC-DAB procedure [73]. The microphotograph, expanded in the inset, reveals the track of the micropipette and multiunit labeling at the recording site (black arrowhead). (B1–B3): Three 500-ms episodes of paired ECoG-LFP recordings showing either simultaneous γ bouts (B1, gray area), a γ bout only in the ECoG (B2, gray area), or a γ bout only in the LFP (B3, gray area). On the top, the numbers give the probability of occurrence of each pattern (semi-quantitative analysis with N>100 from 3 experiments). (C): 500-ms episodes under control (vehicle), ketamine (∼15 minutes after subcutaneous injection of 2.5 mg/kg), then MK-801 (∼30 minutes after subcutaneous injection of 0.1 mg/kg) conditions. The intracortical LFP and the surface ECoG are recorded with a bandpass of 10–100 Hz. (D): Each chart shows the evolution of the γ power (FFT) in the ECoG (left) and intracortical LFP (right) during a 12-s period under control (saline; thick line) and ketamine (2.5 mg/kg; dotted line) conditions. Note that ketamine simultaneously increases the γ power in both the intracortical LFP and the surface ECoG. (E): Average cross-correlation histogram between the ECoG (reference) and the underlying LFP (means of 7×200 ms). Note that ketamine increases the γ rhythmicity (arrowheads) simultaneously in the surface ECoG and the related intracortical LFP. (F): Pearson's correlation (Pcc) of continuous FFT values (N>100) in γ power. As indexed by degree of linear correlation, the coherence in γ power between ECoG and LFP increases after ketamine injection (t test of Bonferroni; p). (G) Pearson's correlation coefficients (Pcc) under different conditions (control, ketamine [∼15 min after 2.5 then 5 mg/kg subcutaneous injection], post-ketamine [>45 min], and MK-801 [∼30 min after 0.1 mg/kg subcutaneous injection]) from 6 experiments (asterisks when Bonferroni p<0.05; ns, non-significant). Ketamine exerts an apparent dose-effect on the γ power coherence.

**Figure 7:**
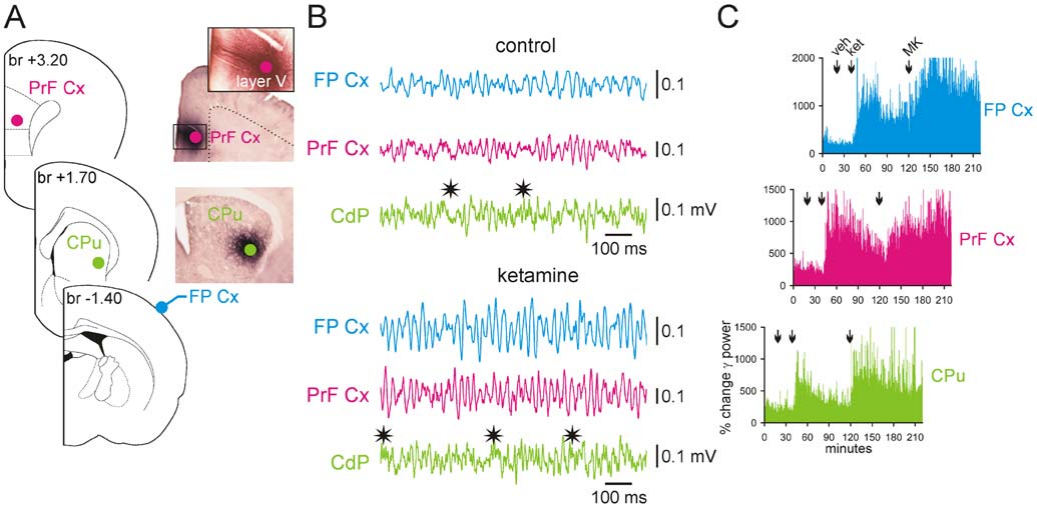
Ketamine or MK-801 increases the amount of γ oscillations in the prefrontal cortex and striatum. These triple recordings were made under fentanyl marcosis. (A): Schematic representation (modified from Paxinos and Watson, 1998) of the recording LFP sites. The stereotaxic position of each coronal plane is given in mm relative to bregma (br). The microphotographs show the location of the recording sites, which was proved following extracellular application of Neurobiotin made at the end of the recording session. The upper-right inset shows, at higher magnification, the apical dendrites of pyramidal neurons of layer V, the location of the recording site (multiunit labeling). (B): 1-s episodes of LFP (bandpass: 10–200 Hz) recorded ∼15 min after subcutaneous injection of vehicle (saline, 1 ml/kg = control) then ∼20 min after injection of ketamine (5 mg/kg). Note that systemic injection of ketamine increases the amplitude of γ oscillations in all recorded structures. High-frequency (81–160 Hz) oscillations are also increased in the striatum and prefrontal cortex (asterisks). (C): Simultaneous % changes in γ power at the three recording sites after subcutaneous injections of vehicle (veh; 1 ml/kg), ketamine (ket; 5 mg/kg) then of MK-801 (MK; 0.08 mg/kg). CPu, caudate putamen; PrF Cx, prefrontal cortex; FP Cx, frontoparietal cortex.

In an attempt to assess the contribution of intracortical networks in the NMDAr antagonist-induced γ hyperactivity recorded in the surface ECoG, it was recorded simultaneously with an underlying intracortical extracellular LFP in layer V under sedated state (Fig. 6A). The present study demonstrates that baseline γ oscillations recorded in behaving or sedated rats share similar properties and are similarly affected by non-competitive NMDAr antagonists. From the raw paired ECoG-LFP recordings obtained under neuroleptanalgesia or narcosis, 3 cases were noticeable: simultaneous, near-synchronized (phase lag: ±6 ms) bouts of γ oscillations in both the ECoG and the LFP (Fig. 6B1); bouts of γ oscillations in either the ECoG, or the LFP (Fig. 6B2,B3). The intrinsic frequency of γ oscillations in the LFP was not significantly different to that of the surface ECoG (respectively: 32.3±0.9 Hz and 34.7±1.2 Hz; N = 25; p>0.05). Ketamine and MK-801 significantly increased to the same proportion the power of γ oscillations in both the surface ECoG and in the underlying intracortical LFP (Fig. 6D). Cross-correlation histograms revealed a noticeable correlation increase between the surface ECoG and the underlying intracortical LFP after injection of ketamine or MK-801 (Fig. 6E), with an oscillation strength about tenfold lower than the full scale ( = 1) and with a variable time lag (0–10 ms). The Pearson's correlation indicated that the degree of linear relationship between ECoG and associated LFP FFT values was relatively small (<0.2) and was significantly increased (up to 0.2–0.6) after systemic injection of ketamine or MK-801 (Fig. 6F,G). Similar correlation degrees were observed with LFP recordings in parietal, frontal and prefrontal cortical areas, that is, at different distances from the surface ECoG (not shown). Furthermore, two adjacent sub-networks (e.g., layer V and VI – 200 µm apart - in a putative column) of the frontoparietal cortex individually had a similar relationship with the related surface ECoG but, on the other hand, they could generate highly correlated ongoing γ oscillations (not shown). These experiments suggest that the surface ECoG reflects the integration of ongoing γ oscillations generated either from a large-scale cortical network, or from multiple cortical networks. High-resolution studies are required to understand the vertical and horizontal spatio-temporal dynamics of the generation of ongoing rhythmic γ waves in a given part of the neocortex. Also, ketamine and MK-801 dramatically increased the amount of γ oscillations in the prefrontal cortex with a pattern similar to that recorded in the frontoparietal cortex (Fig. 7). The average intrinsic frequency of γ oscillations in the frontoparietal and prefrontal cortices is closely similar (in the same experiment: 38.4±0.8 Hz and 40.5±0.7 Hz, respectively; p>0.05).

To examine the possible contribution of subcortical systems in the generation of ketamine-induced aberrant γ activity, 1 or 2 distant subcortical sites were recorded along with the frontoparietal surface ECoG (minimum 2–3 rats/recording site). We recorded in structures that are known to generate, under normal conditions, γ oscillations like the accumbens [21], amygdala [22], basalis [23], hippocampus [24] and thalamus [25], [26]. The striatum was also recorded since it receives projections from the frontoparietal cortex [27] and generates γ oscillations during movement initiation [28]. In the striatum of sedated, narcotized or neuroleptanalgesied rats, ketamine and MK-801 increased the amount of γ oscillations (Fig. 7). Nevertheless, the pattern of striatal rhythmic γ waves was not as stereotyped as that displayed by the frontoparietal and prefrontal cortices, probably because of the simultaneous occurrence of higher-frequency (81–160 Hz) oscillations in the striatum (Fig. 7B).

**Figure 8:**
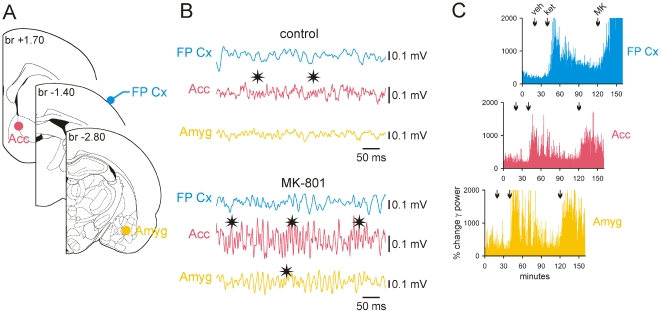
Ketamine or MK-801 increases the amount of γ (30–80 Hz) and higher frequency (81–160 Hz) oscillations in the accumbens and amygdala. These triple recordings were made under fentanyl marcosis. (A): Schematic representation (modified from Paxinos and Watson, 1998) of the recording LFP sites. The location of the recording sites was proved following extracellular application of Neurobiotin made at the end of the recording session. The stereotaxic position of each coronal plane is given in mm relative to bregma (br). (B): 400-ms episodes of LFP (bandpass: 10–200 Hz) recorded ∼15 min after subcutaneous injection of vehicle (saline, 1 ml/kg = control) then ∼30 min after injection of MK-801 (0.08 mg/kg). Note that systemic injection of MK-801 increases the amplitude of γ oscillations in all recorded structures. In the accumbens and amygdala, the amount of high-frequency oscillations (asterisks) is also increased after MK-801 injection. Gamma and higher frequency oscillations are distinguishable. (C): Simultaneous % changes in γ power at the three recording sites after subcutaneous injections of vehicle (veh; 1 ml/kg), ketamine (ket; 5 mg/kg) then of MK-801 (MK; 0.08 mg/kg). Acc, accumbens; Amyg, amygdala; FP Cx, frontoparietal cortex.

**Figure 9:**
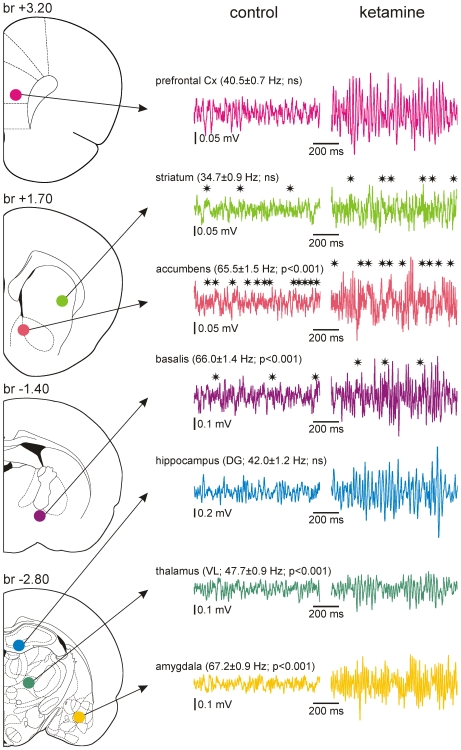
Ketamine increases the amount of ongoing γ oscillations in the prefrontal cortex and subcortical structures. Left panel: Schematic representation (modified from Paxinos and Watson, 1998) of the recording LFP sites. The location of the recording sites was proved following extracellular application of Neurobiotin made at the end of the recording session. The stereotaxic position of each coronal plane is given in mm relative to bregma (br). Right panel: 1-s episodes of LFP recorded (bandpass: 10–200 Hz) under fentanyl sedated state ∼15 min after subcutaneous injection of vehicle (saline, 1 ml/kg = control) then ∼15 min after injection of ketamine (5 mg/kg). Note that systemic injection of ketamine increases the amplitude of γ (30–80 Hz) oscillations in all recorded structures (1–2 subcortical sites recorded simultaneously with the frontoparietal surface ECoG). In the accumbens, basalis and striatum, the amount of high-frequency oscillations (81–160 Hz; asterisks) is also increased after ketamine injection. For each recording site, the average internal frequency of γ oscillation (±sem) is determined from 25 100-ms episodes. This frequency is compared with that measured in the corresponding frontoparietal ECoG (Student t test; ns, non-significant). The internal frequency of γ oscillations recorded in the frontoparietal cortex varies from 30 to 50 Hz (40.2±0.7 Hz). The measured average frequencies do not include high-frequency oscillations that were recorded especially in the accumbens, basalis and striatum (asterisks). Cx, cortex; DG, dendate gyrus; VL, ventral lateral.

Systemic injection of ketamine or MK-801 dramatically increased the amount of background γ oscillations in many other regions, including the zona incerta (Fig. S3), the substantia innominata and lateral hypothalamus (not shown). For instance, both the amygdala and the accumbens exhibited γ oscillations with similar properties (time of occurrence, duration and amplitude) but dissimilar to those recorded simultaneously in the frontoparietal ECoG (Fig. 8B). More specifically, the intrinsic frequency of these two deep limbic structures (67.2±0.9 Hz and 65.5±1.5 Hz, respectively; these two values are not significantly different: p>0.05) was significantly higher than that of the associated cortical γ oscillations (40.2±0.7 Hz; Student t test, p<0.001) (Fig. 9). In addition, the accumbens and amygdala simultaneously displayed episodes of high frequency (81–160 Hz) oscillations, which were also increased in power after ketamine or MK-801 injection (Fig. 8, Fig. 9). The observed differences in γ properties between the cortical and these two deep limbic structures do not support the hypothesis that bursts of γ oscillations recorded in the cortex were volume conducted from the accumbens and amygdala. On the other hand, these recordings consistently demonstrated that these two structures roughly displayed the same pattern of γ and higher frequency oscillations, especially after ketamine or MK-801 injection (Fig. 8B), probably because of their anatomical connections. High-resolution anatomo-functional studies are required to understand whether ketamine-induced γ hyperactivity first started in the accumbens, amygdala, or simultaneously in both structures.

Most nuclei of the thalamus, including the associative (e.g., posterior group), limbic, motor and sensory nuclei that are related to the frontoparietal cortex, increased their basal γ activity after injection of ketamine or MK-801 (Fig. S3). Ketamine-induced increases in rhythmic γ waves were also recorded in the thalamic reticular nucleus (not shown). Further studies are however required to know whether or not NMDAr antagonists have a primary site (cortical or thalamic) of action in corticothalamic systems.

## Discussion

The present study demonstrates 1) that a single systemic injection of a low-dose of ketamine produces temporally correlated hyperlocomotion and generalized persistent aberrant γ oscillations; 2) that these pathophysiological γ waves are not caused by conscious sensorimotor processing underlying hyperlocomotion-related brain state; 3) that the ECoG mainly reflects γ oscillations recorded in intracortical networks; 4) that they occur all over the cerebral cortex and in multiple subcortical structures, including sensory, motor, limbic and associative/cognitive systems, and 5) that NMDAr antagonist-induced ongoing γ hyperactivities can be recorded under diverse consciousness states.

### 1. NMDAr antagonist-induced γ hyperactivities are not caused by conscious sensorimotor processing underlying abnormal motor behavior

Non-competitive NMDAr antagonist-induced persistent γ hyperactivity was recorded under diverse brain states: in conscious freely moving rats and in deeply modified states of consciousness. They were obtained during continuous administration of an anesthetic (urethane or pentobarbital) or sedative substances (fentanyl and haloperidol). Therefore our results demonstrate that the frontoparietal (or sensorimotor) γ hyperactivity and hyperlocomotion are two independent effects of administration of low-dose (<5 mg/kg) of non-competitive NMDAr antagonists. Our experiments made in anesthetized and sedated rats thus provide strong evidence that the NMDAr antagonist-induced γ hyperactivity was not the consequence of conscious sensorimotor processing, nor is it directly linked to the motor effects seen in freely moving rats (i.e. ataxic-like behavior and hyperlocomotion), which may not occur to the same extent in humans with ketamine-induced psychosis. Furthermore, our multisite recordings have demonstrated that the NMDAr antagonist-induced γ hyperactivity is a generalized phenomenon, not specific to sensorimotor systems.

### 2. Is hyperlocomotion-related brain state a psychotic state?

The non-anesthetic low-doses of ketamine that were used here to produce hyperlocomotion and aberrant γ oscillations are an order of magnitude lower and less toxic than those used in earlier studies [29]–[32]. Also, the doses of ketamine used in the present work (also see Pinault, 2008) are similar to those that induce cognitive deficits in humans [8]–[11]. In rodents, such a dose induces sensory gating impairment [33] and memory deficit [34], [35]. Of importance, the kinetics of the ketamine action on behavior and basal γ oscillations are quite consistent with plasma and brain half-life measured following injection of ketamine [36]. These recent findings, and the present demonstration that hyperlocomotion and γ hyperactivity on EEG, are two independent effects of low-dose ketamine administration leave open the question about the nature of the ketamine-induced abnormal brain state associated with γ hyperactivity in rodents and their possible relevance to psychotic symptoms in humans. Electroclinical data suggest that hypersynchronized γ oscillations may be associated with hallucinations (see below). Moreover, ketamine is also used as a recreative substance causing psychological dissociation [37]. However, further studies that investigate the effect of anti-psychotic medications would be helpful to more firmly establish the link between this electrophysiological finding and psychosis.

### 3. Ketamine and MK-801 produce generalized and persistent aberrant γ oscillations in cortical and subcortical structures

Our multiple recordings carried out in free-moving, sedated and anesthetized rats, have demonstrated that non-competitive NMDAr antagonists lead to a significant power increase in ongoing γ oscillations simultaneously all over the cerebral cortex, at least in the prefrontal, frontal, parietal and occipital areas, and in multiple subcortical structures, including the amygdala, accumbens, striatum, basalis, hippocampus and thalamus. Of importance, we have demonstrated that the experimental conditions slightly modify the intrinsic properties of ongoing γ oscillations but did not change the consistent effect of ketamine and MK-801 on the γ power. Moreover, in free-moving rats, a low-dose of ketamine (6 mg/kg, sc) induces γ power increases in the hippocampus, deficits in sensory gating and hyperlocomotion [33]. In conclusion, our findings demonstrate that the psychotomimetics ketamine and MK-801 act, directly or indirectly, in almost all brain networks that support the dynamics of limbic, sensorimotor, and cognitive processes. Further studies are, however, required to know whether or not this apparent generalized neurophysiological effect of non-competitive NMDAr antagonists primarily involves a preferential structure or system.

The present findings support the hypothesis that ongoing γ oscillations recorded in the surface ECoG mainly mirror rhythmic γ waves generated by intracortical networks. Moreover, our multisite recordings show that most of ECoG γ oscillations cannot be volume-conducted from subcortical regions that display different γ frequency bands and patterns. For instance, the accumbens, basalis and amygdala display γ waves at higher frequencies than the cerebral cortex (∼65 Hz vs ∼40 Hz). Furthermore, the accumbens, striatum and amygdala in addition exhibit higher-frequency (81–160 Hz) oscillations, which were not reflected in the frontoparietal ECoG and which were significantly increased after ketamine or MK-801 injection. Moreover, a previous study conducted in freely moving rats demonstrated that ketamine significantly increases the amount of high-frequency oscillations in the accumbens [29]. Our findings further demonstrate that two distinct systems can simultaneously exhibit ongoing γ waves at quite different frequencies. More specifically, on the one hand the sensorimotor cortex, thalamus and striatum and, on the other hand, limbic structures display γ waves at ∼35–45 Hz and 60–80 Hz, respectively. Also, both the prefrontal cortex and the hippocampus, which are connected, generate ongoing γ waves at ∼40 Hz.

One may wonder i) what does the acutely ketamine-treated rodent model, and ii) whether, in schizophrenic patients, the whole brain or a given system is, at some stages, in a state associated with generalized or localized hypersynchronized γ oscillations. Further experimental and clinical studies are required to better understand i) the mechanisms underlying the psychotomimetic action of ketamine at low-doses, and ii) the spatio-temporal patterns of ongoing γ oscillations in healthy subjects and schizophrenic patients.

### 4. Possible mechanisms underlying NMDAr antagonist-induced generalized, persistent and aberrant γ noise

It is assumed that the γ power recorded in a given structure reflects the synchronization of pools of neurons oscillating at more or less the same frequency. Since two distinct structures can oscillate at different γ frequencies, it is reasonable to say that the generalized action of NMDAr antagonists induced γ hypersynchronies or aberrant γ noise.

Ketamine-induced, persistent and aberrant γ noise represents a general phenomenon since it can be recorded in free-moving rats, under narcosis, neuroleptanalgesia and urethane-anesthesia and, to a lesser extent, under pentobarbital anesthesia. This strongly indicates that ongoing γ oscillations and their increase by NMDAr antagonists are underlain by similar mechanisms. Interestingly, under pentobarbital, the amount and persistent character of γ oscillations was significantly lower than those recorded under the other conditions. This might be explained by the barbiturate-induced prolongation of the decay time of GABA(A)r-mediated IPSPs [38].

The present study further demonstrates that NMDAr antagonist-induced γ hypersynchronies simultaneously occur in cortical and subcortical structures, at least in those having cellular and network properties to generate γ oscillations under physiological conditions (see references in results). The current literature suggests that γ oscillations may be a biomarker of the collective activity of networks of GABAergic interneurons [39]–[41]. It is worth mentioning that corticothalamic systems are composed of neurons with pacemaker properties in the γ frequency band [25], [26], [42], [43], which could interfere with oscillating cortical networks.

The possible mechanisms underlying NMDAr antagonist-induced γ hypersynchronies are unknown [44]. Multi-unit recordings in the prefrontal cortex of control and MK-801-treated awaked rats revealed opposite effects on the firing of fast spiking and regular spiking neurons [45]. These data suggest that disinhibition of GABAergic interneurons that control the firing of pyramidal neurons might be secondary to NMDAr hypofunction, leading to hyperexcitation of glutamatergic neurons. Such disinhibition might be due to a direct blockade of NMDAr in interneurons and/or to a blockade of presynaptic, NMDA-dependent release of glutamate [46], [47]. The action of ketamine might also involve α-amino-3-hydroxy-5-methylisoxazole-4-propionic acid (AMPA) receptor throughput [48].

Further studies are required to determine whether the pathophysiological γ hypersynchronies result from a direct or downstream effect of the non-competitive NMDAr antagonists. Supposing that the effect is direct, it would be important to identify the primary site of action of such antagonists in neural networks, which are mainly composed of GABAergic, glutamatergic neurons, and glial cells. Indeed, NMDA can activate not only neurons but also glial cells [49]. The latter elements play a great role in neuronal synchrony, for instance via extrasynaptic NMDAr [50]. It is also tempting to put forward the hypothesis that ketamine blocks astrocytic NMDAr and subsequently the astrocytic release of D-serine, a potential target for a new generation of antipsychotics [51].

We cannot exclude that the psychotomimetics ketamine and MK-801 also bind at other receptors, especially on dopaminergic and serotoninergic receptors [52], [53]. In our previous study, the slight but statistically significant γ hyperactivity following activation of dopaminergic receptors in conscious rats does not explain the transient important effect induced by these psychotomimetics on the generalized γ hypersynchronies [12]. The fact that the power of ongoing γ oscillations is higher under anesthesia (also see Vanderwolf, 2000) suggests that the normal behavior and consciousness exert a certain “inhibitory control” in the generation of ongoing γ oscillations.

It is worth noting that, in humans, ketamine also interacts with GABA(A)α2 receptors, at least in the dorso-medial prefrontal cortex [54]. Interestingly, GABA(A)α2 receptor agonists improve cognition and increase the power of induced (not time-locked to stimulus; also see below) γ oscillations in the frontal cortex of schizophrenic patients [55].

### 5. Functional impact of ongoing pathophysiological γ noise

Here it has been demonstrated that, in rodents, the psychotomimetics ketamine and MK-801 acutely disturb the brain state characterized by generalized ongoing γ hypersynchronies in cortical and subcortical, sensorimotor, limbic and cognitive networks. All these systems are affected in patients with schizophrenia [56]. In humans, non-competitive NMDAr antagonists induce cognitive impairments and schizophrenia-like symptoms (see references in introduction). So the present findings raise important issues relevant to the understanding of the link between ketamine-induced generalized and persistent γ hyperactivity and the symptoms and abnormalities of γ oscillations in schizophrenia. One major issue is the type of γ oscillations in question. At least four types of γ activity should be considered: 1) Spontaneously-occurring or ongoing γ oscillations (or normal γ noise), which is usually recorded during desynchronized state of the electroencephalogram (the current study, [12], [57], [58]), 2) γ steady-state response [59], 3) Evoked γ response, which is phase-locked to the stimulus onset [60], and 4) Cognition or perception-related induced coherent synchronized γ oscillations [61], [62], [58]. In healthy subjects or patients with schizophrenia, it is the latter type that is usually referred to, which represents high-order task-related ephemeral and synchronized γ oscillations. The γ steady-state response is also altered in schizophrenic patients [63]. One may thus predict that ongoing γ noise may, under certain circumstances, modulate steady state, evoked and/or induced rhythmic γ waves (see below).

The research conducted so far is conflicting regarding the relation between γ oscillations and schizophrenic symptoms. Increases and decreases in γ oscillations (power and frequency) have been reported in schizophrenic patients during a given mental task [64], [65], [66], [67]. The human literature in particular is confounded by technical difficulties, including the head regions of the recordings, the nature of the symptoms, and on the effects of anti-psychotic drug treatment. The purpose of this study was not to address the relationship between abnormal γ activity on the EEG and psychotic symptoms, but rather to address the relationship between the γ hyperactivity and hyperlocomotion induced by low dose ketamine. The relevance between these and psychosis is inferential, in that equivalent doses of ketamine are known to induce psychotic symptoms in humans.

In an attempt to make a link of these clinical data with our findings, it is tempting to put forward a prediction. In the present study, we have demonstrated that non-competitive NMDAr antagonists lead to ongoing, generalized and persistent, aberrant γ oscillations. This indicates that the normal, low-amplitude γ noise is pharmacologically metamorphosed into an aberrant, high-amplitude γ noise. In humans, if such abnormal γ noise does exist, it would drown the transient (evoked and/or induced) γ responses, for instance the cognition-related coherent γ synchrony. In other words, such aberrant noise would decrease or annihilate the γ signal-to- γ noise ratio of task-related transient γ synchronies. So if the aberrant γ noise disrupts ephemeral synchronized γ oscillations, which are thought to contribute to high-order brain operations, it would cause cognitive dysfunction. In other words, in schizophrenic patients ongoing γ hypersynchrony would disrupt functional (e.g., sensorimotor) integration in highly distributed systems and disintegrate psychic processes. This prediction may be applied to perception- and working memory-related induced γ oscillations [68]. However, validation of this prediction in humans requires specific clinical experiments to be performed. Our prediction is indirectly supported by a clinical study showing that GABA(A)α2 agonists improve cognition and increase the power of induced γ oscillations in schizophrenic patients [55].

Does abnormal γ noise (ongoing hypersynchronized γ oscillations) exist in schizophrenic patients? Is ongoing γ hyperactivity related to positive and/or negative symptoms? Although there is no conclusive evidence that ongoing, generalized or localized, aberrant γ noise is a hallmark of schizophrenia, increased γ synchrony has been recorded in patients during somatic and visual hallucinations [69], [70], [71], [66].

## Materials and Methods

Forty-six adult male Wistar rats (250–350 g) were used in accordance with Australian and European guidelines (directive 86/609/EEC) and were approved by the Animal Ethics Committees of Medicine (RMH) University of Melbourne (#0701821) and of University Louis Pasteur/University of Strasbourg (CREMEAS, AL/03/15/12/05). All efforts were made to avoid animal suffering and use the minimal number of animals to produce reliable data. Rats were maintained in 12-h light/dark cycle, illuminated from 07:00 to 19:00 h with food and water available ad libitum. Experiments in freely moving rats were performed during the light phase (09:00–17:00 h), and those in anesthetized unconscious rats were carried out in 09:00–20:00 h.

### Drugs

Ketamine was obtained from Merial (Lyon, France) and from Troy Laboratories PTY Limited (NSW, Australia), MK-801 and D-Tubocurarine chloride from Sigma-Aldrich (Saint-Quentin Fallavier, France; NSW, Australia), pentobarbital from Sanofi (Libourne, France), fentanyl and haldol from Janssen (Boulogne-Billancourt, France). Xylazine was obtained from Sigma-Aldrich PTY. LTD (NSW, Australia). All drugs were dissolved in physiological saline (NaCl, 0.9%).

### Surgery for chronic experiments

Rats (N = 8) were anesthetized with xylazine (10 mg/kg, i.p.) and ketamine (75 mg/kg, i.p.) and positioned in a stereotaxic frame. A single midline incision was made over the scalp and six holes were drilled through the skull for stereotaxic [72] implantation of recording brass electrodes (2 mm anterior and 2 mm lateral to bregma bilaterally (active electrodes); 2 mm posterior and 2 mm lateral to bregma bilaterally (ground electrodes); and 2 mm posterior and 2 mm lateral to lambda bilaterally (reference electrodes). The electrodes were screwed into the skull without breaching the dura, and dental cement was applied to fix the electrodes in place. The animals were then placed in separate cages for 7-days recovery with food and water ad libitum prior to the pharmacological experiments.

In a second series of chronic experiments, two stainless steel screws were implanted under stereotaxic guidance extradurally either over the frontal (or motor; from bregma: 1 mm anterior and 2 mm lateral) and parietal (or somatosensory; −1 mm posterior and 4 mm lateral) cortices (4 rats), or over the frontal and occipital (or visual; −5 mm posterior and 2 mm lateral) cortices (3 rats). Surgery was made under deep anesthesia (pentobarbital: 40 mg/kg, i.p. and ketamine: 50 mg/kg, i.m.). Two additional screws were fixed in the frontal bone for ground connection and in the skull over the cerebellum for reference. The screws were connected to a subminiature connector fixed to the skull with dental cement.

### Surgery and experimental conditions for acute experiments

Surgical procedures were made under deep general anesthesia with pentobarbital (40 mg/kg, i.p.) and ketamine (50 mg/kg, i.m.) and under stereotaxic conditions. For the recording session, this anesthesia was discontinued and the rat was maintained under one of these four states: 1) deep anesthesia induced by urethane (1.5 g/kg, i.p.; 4 rats); 2) deep barbiturate–fentanyl anesthesia (7 rats) induced by continuous intravenous injection (0.5 ml/h) of the following mixture (quantity given per hour for a rat of 300 g): fentanyl (1 µg), pentobarbital (3.5–8.2 mg) and glucose (25 mg); 3) neuroleptanalgesia (12 rats) produced by continuous intravenous injection of the following mixture (quantity given per hour for a rat of 300 g): fentanyl (1 µg), haldol (100 µg) and glucose (25 mg); 4) Sedated narcotized state (8 rats) induced by continuous intravenous injection of the following mixture (quantity given per hour for a rat of 300 g): fentanyl (2 µg) and glucose (25 mg). Muscle rigidity and tremors were blocked with intravenous administration of D-Tubocurarine chloride (0.4 mg/hr). The rats were artificially ventilated in the pressure mode (8–12 cm H_2_O; 60 bpm) using an O_2_-enriched gas mixture (50% air-50% O_2_). The rat's rectal temperature was maintained at its physiological level (37–38.3°C) using a thermoregulated blanket. The electrocorticogram (ECoG) and the heart rate were also under continuous monitoring to maintain a steady depth of anesthesia or sedation either by giving a bolus or adjusting the injection rate of the anesthetizing or sedating mixture. The depth of anesthesia was ascertained by the occurrence of slow waves in the ECoG. Recording sessions started 3 hours after state induction. Local anesthetic (lidocaine, 2%) was infiltrated in all surgical wounds every 2 hours.

### ECoG and locomotion in freely moving rats

On the day of testing, rats were brought into the behavioral testing facility 30 minutes prior to experimentation to allow habituation to the environment. They were then individually placed into an open arena (1 m diameter) with the recording electrodes attached to a cable suspended from the ceiling to record the ECoG. The rat was allowed to explore the arena for 30 minutes after which they were subcutaneously (sc) injected with either ketamine (2.5 and 5 mg/kg), MK-801 (0.08 and 0.16 mg/kg), or vehicle (0.9% NaCl) and returned to the arena for a further 90 minutes. This procedure was repeated on subsequent days with a different drug treatment until each rat had received each dose of each drug. During the 120-minutes recording period, the animal's locomotor activity was continuously video-tracked and objectively assessed using Ethovision Software (Noldus®, Netherlands). The total distance travelled (i.e. locomotion) was calculated every 2 seconds during the entire recording session.

### ECoG and local field potential (LFP) recordings, and drug injection under acute conditions

The monopolar frontoparietal ECoG was recorded with silver wire (diameter: 150 µm) insulated with teflon (inserted in the bone without contact with the dura mater; see Fig. 4A) and connected to an ultralow noise amplifier (AI 402, x50; Axon Instruments, subsidiary of Molecular Devices, Sunnyvale, California, USA). The reference electrode (set to ground) was inserted into the occipital crest). Multiple subcortical LFP recordings were obtained with glass micropipettes filled with ACSF-Neurobiotin (1%). Ketamine or MK-801 and the corresponding vehicle (0.9% NaCl) were subcutaneously administered (1 ml/kg) or intravenously (maximal injection volume = 0.25 ml; injection time: ∼2 min; see Fig. S2 and S3). At the end of the recording session, extracellular iontophoresis of Neurobiotin was achieved with positive current pulses (200 ms on/200 ms off; +600 nA during 5 min) delivered by a microiontophoresis current generator (SYS-260; WPI, Sarasota, FL, USA). After a survival time (<60 min) the animals were euthanized with a lethal dose of pentobarbital; their brain were fixed transcardially with 4% paraformaldehyde, removed and processed for histology to reveal the neuronal tracer (Neurobiotin) for localization and identification of the recording sites using standard procedures.

### Signal conditioning

In freely-moving rats, the bilateral ECoG was processed using an electro-encephalographic hardware/software (Chart V 3.5, ADI Instruments, Mac Lab) with a bandpass of 1–1000 Hz and digitized at 1.0 kHz. The line power 50 Hz noise was eliminated from the signal using selective filters (Humbugs; Digitimer, Letchworth Garden City, UK). In acute experiments, the ECoG and LFP were processed with a bandpass of 0.1–800 Hz and digitized at 10 kHz (Clampex, v7, Axon Instruments).

### Quantitative and statistical data analyses

ECoG data from freely moving rats were analyzed using NEUROSCAN® software (Compumedics, Melbourne, Australia). Using Fast Fourier Transformations (FFT), average power in the γ frequency band (30–80 Hz) was determined for each 2.05 sec epoch for the entire duration of the recording period. For both γ power and locomotor activity data, the values obtained during the 30-minutes pre-injection period were averaged for each individual animal, and all recorded values then expressed as a percent change of this response. Two endpoints were quantified for statistical comparison for both the γ power analyses and the locomotor activity measurements: the peak drug response and the total drug response as assessed by measuring the area under the curve (AUC). Peak responses were compared using one-way ANOVA with repeated measures (dose of drug). AUC measures were calculated by determining at which times the trace acquired from the highest drug dose increased >2 standard deviations above the vehicle-treated trace. Total area was then calculated during these periods. For ketamine-treated rats, this represented the period starting at the time of drug injection and ceasing after 30 minutes, and for rats treated with MK-801, this period began 10 minutes after the injection and persisted until the end of the recording period. For correlation analyses, data were split into three 30-min blocks after drug treatment. Group mean γ power measures at each two minute time point were related with the corresponding group mean locomotor activity, and Spearman's correlation coefficients (r) were then calculated for each time window for each drug. For statistical analyses, correlation coefficients were calculated for each individual rat following each drug for the same 30-minute blocks, and group means calculated for each time window. Statistical comparison of the mean correlation coefficients for each drug was then performed using one-way ANOVA with repeated measures for each time window, using Dunnett's post-hoc analysis if appropriate comparing ketamine and MK-801 to vehicle. Data were analyzed using Statistica software® (Tulsa, OK) and statistical significance was set at p<0.05 in all cases.

The FFT of ECoG recordings from acute experiments were computed using DataWave softwares (SciWorks, v4, DataWave Technologies, Berthoud, CO, USA). Spectral analysis was based on 1.6-s epochs, with a resolution of 0.610 Hz and with a hamming window to prevent spectral leakage. The 50 Hz values were discarded to avoid contamination from possible AC noise. The sum of the 30–49 Hz and 51–80 Hz FFT values gave the total power of γ oscillations (30–80 Hz). The frequency at maximal power was also extracted. Thereby, properties of γ oscillations recorded before and after the administration of a given agent (vehicle or substance) could be compared. They were evaluated for statistical significance using Student's t-test, the significance level being set to 0.05.

The degree of linear relationship between continuous FFT values (N>100) of γ oscillations recorded in two regions (surface ECoG and underlying intracortical LFP) was assessed using the Pearson's correlation (implemented with Bonferroni probability). Thereby, the Pearson's correlation coefficient gave an index of the coherence of γ oscillations between these two regions.

Data are presented as means±s.e.m.

## Supporting Information

Figure S1 Pattern of ECoG γ oscillations under different experimental conditions. Typical 1-sec ECoG episodes under drug-free awaked condition (FREE), fentanyl-haldol neuroleptanalgesia (SEDATION), urethane-anesthesia and pentobarbital-fentanyl (PENTO) anesthesia.
(0.03 MB PDF)

Figure S2 Ketamine or MK-801 dose-dependently increases the power of ongoing γ oscillations under neuroleptanalgesia. (A), (B1) and (B2) are from three experiments under fentanyl-haldol neuroleptanalgesia. (A): Changes in γ power during a full recording session under 3 different conditions, vehicle (NaCl), ketamine, and MK-801 (sc, subcutaneous injection). (B1 or B2): Changes in γ power during a recording session, during which the rat received intravenous (iv) injections (increasing doses; arrows) of ketamine (B1) or MK-801 (B2).
(0.06 MB PDF)

Figure S3 A single intravenous injection of ketamine (0.5 mg/kg) quickly increases the power of γ oscillations in the frontoparietal cortex, hippocampus, thalamus and zona incerta. (A): Experimental design (dorsal and coronal views): In these experiments (N = 3), the frontoparietal (FP) ECoG (or FP cx) and the hippocampal (dentate gyrus or DG) LFP recordings were permanent. On the other hand, a Neurobiotin-ACSF-filled micropipette (tip diameter: 3–7 µm) was moved down in subcortical structures, including the thalamus (Th) and zona incerta (ZI). At the end of the recording session, the neuronal tracer is applied using extracellular iontophoresis (+600 nA, 200 ms on, 200 ms off, for 10 min). The tracer is revealed using a standard ABC-DAB procedure (Pinault, 1996). The microphotographs reveal the location of the recording sites (black spots). (B): The left and right panels show % change in γ power measured from two successive triple recording sessions, FPcx-DG-Th and FPcx-DG-ZI. The second intravenous injection of ketamine was made more than 2 hours after the first injection. Note that ketamine increases the γ power simultaneously at all recording sites. (C): The charts show the quick increase in γ oscillations during the first 2 minutes that followed the onset of intravenous injection of ketamine. The grey areas indicate the period during which ketamine was intravenously injected. Each point is the average of 12–15 successive FFT values (±sem) of γ power. Note that, at all recording sites, the γ power starts to increase during ketamine injection.
(0.12 MB PDF)
