# Supplementary material for: NMDA Receptor Hypofunction Leads to Generalized and Persistent Aberrant γ Oscillations Independent of Hyperlocomotion and the State of Consciousness
Source: PLoS One. 2009 Aug 25;4(8):e6755. doi: 10.1371/journal.pone.0006755 (PMC2727800; doi:10.1371/journal.pone.0006755)
Supplement: Figure S1 — Pattern of ECoG γ oscillations under different experimental conditions. Typical 1-sec ECoG episodes under drug-free awaked condition (FREE), fentanyl-haldol neuroleptanalgesia (SEDATION), urethane-anesthesia and pentobarbital-fentanyl (PENTO) anesthesia. (0.03 MB PDF) [file pone.0006755.s001.pdf]

# S1: Pattern of ECoG γ oscillations under different experimental conditions

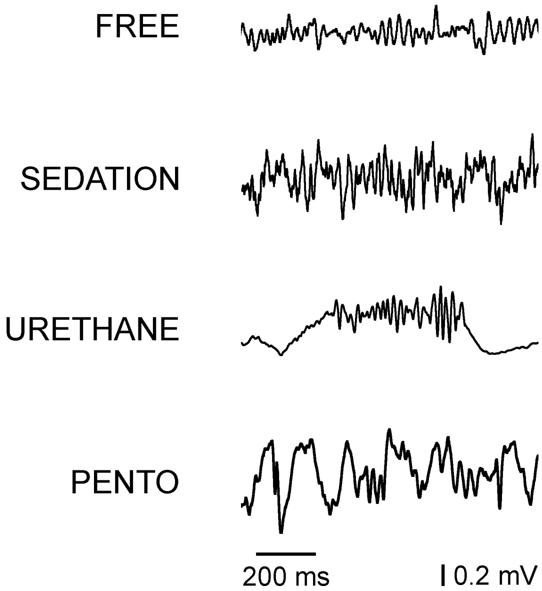


Typical 1-sec ECoG episodes under drug-free awaked condition (FREE), fentanyl-haldol neuroleptanalgesia (SEDATION), urethane-anesthesia and pentobarbital-fentanyl (PENTO) anesthesia.
