# Supplementary material for: NMDA Receptor Hypofunction Leads to Generalized and Persistent Aberrant γ Oscillations Independent of Hyperlocomotion and the State of Consciousness
Source: PLoS One. 2009 Aug 25;4(8):e6755. doi: 10.1371/journal.pone.0006755 (PMC2727800; doi:10.1371/journal.pone.0006755)

S2: Ketamine or MK-801 dose-dependently increases the power of ongoing γ oscillations under neuroleptanalgesia

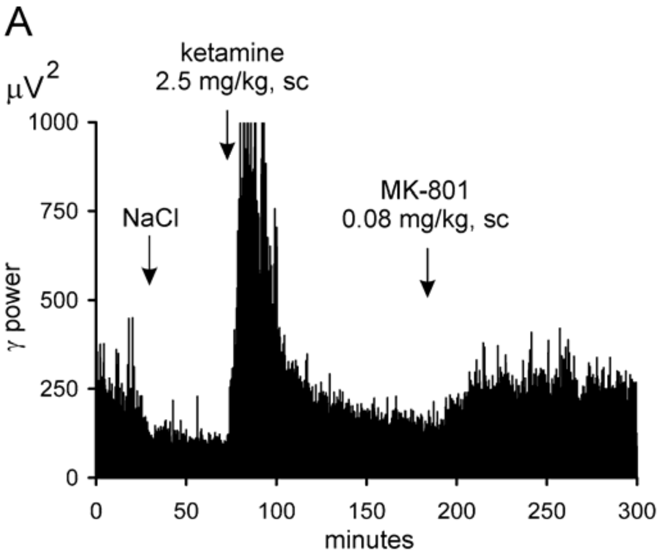

(A), (B1) and (B2) are from three experiments under fentanyl-haldol neuroleptanalgesia. (A): Changes in γ power during a full recording session under 3 different conditions, vehicle (NaCl), ketamine, and MK-801 (sc, subcutaneous injection). (B1 or B2): Changes in γ power during a recording session, during which the rat received intravenous (iv) injections (increasing doses; arrows) of ketamine (B1) or MK-801 (B2).

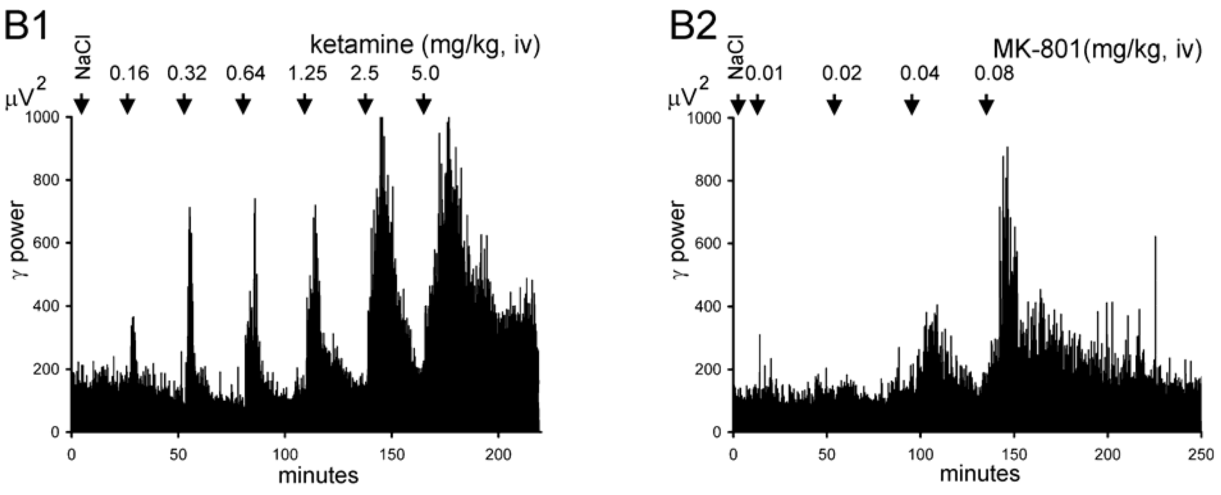

Supplement: Figure S2 — Ketamine or MK-801 dose-dependently increases the power of ongoing γ oscillations under neuroleptanalgesia. (A), (B1) and (B2) are from three experiments under fentanyl-haldol neuroleptanalgesia. (A): Changes in γ power during a full recording session under 3 different conditions, vehicle (NaCl), ketamine, and MK-801 (sc, subcutaneous injection). (B1 or B2): Changes in γ power during a recording session, during which the rat received intravenous (iv) injections (increasing doses; arrows) of ketamine (B1) or MK-801 (B2). (0.06 MB PDF) [file pone.0006755.s002.pdf]
