# Supplementary material for: NMDA Receptor Hypofunction Leads to Generalized and Persistent Aberrant γ Oscillations Independent of Hyperlocomotion and the State of Consciousness
Source: PLoS One. 2009 Aug 25;4(8):e6755. doi: 10.1371/journal.pone.0006755 (PMC2727800; doi:10.1371/journal.pone.0006755)

<u>S3</u>: A single intravenous injection of ketamine (0.5 mg/kg) quickly increases the power of γ oscillations in the frontoparietal cortex, hippocampus, thalamus and zona incerta.

(A): Experimental design (dorsal and coronal views): In these experiments (N=3), the frontoparietal (FP) ECoG (or FP cx) and the hippocampal (dentate gyrus or DG) LFP recordings were permanent. On the other hand, a Neurobiotin-ACSF-filled micropipette (tip diameter: 3-7 μm) was moved down in subcortical structures, including the thalamus (Th) and zona incerta (ZI). At the end of the recording session, the neuronal tracer is applied using extracellular iontophoresis (+600 nA, 200 ms on, 200 ms off, for 10 min). The tracer is revealed using a standard ABC-DAB procedure (Pinault, 1996). The microphotographs reveal the location of the recording sites (black spots). **(B):** The left and right panels show % change in γ power measured from two successive triple recording sessions, FPcx-DG-Th and FPcx-DG-ZI. The second intravenous injection of ketamine was made more than 2 hours after the first injection. Note that ketamine increases the γ power simultaneously at all recording sites. **(C):** The charts show the quick increase in γ oscillations during the first 2 minutes that followed the onset of intravenous injection of ketamine. The grey areas indicate the period during which ketamine was intravenously injected. Each point is the average of 12-15 successive FFT values (±sem) of γ power. Note that, at all recording sites, the γ power starts to increase during ketamine injection.

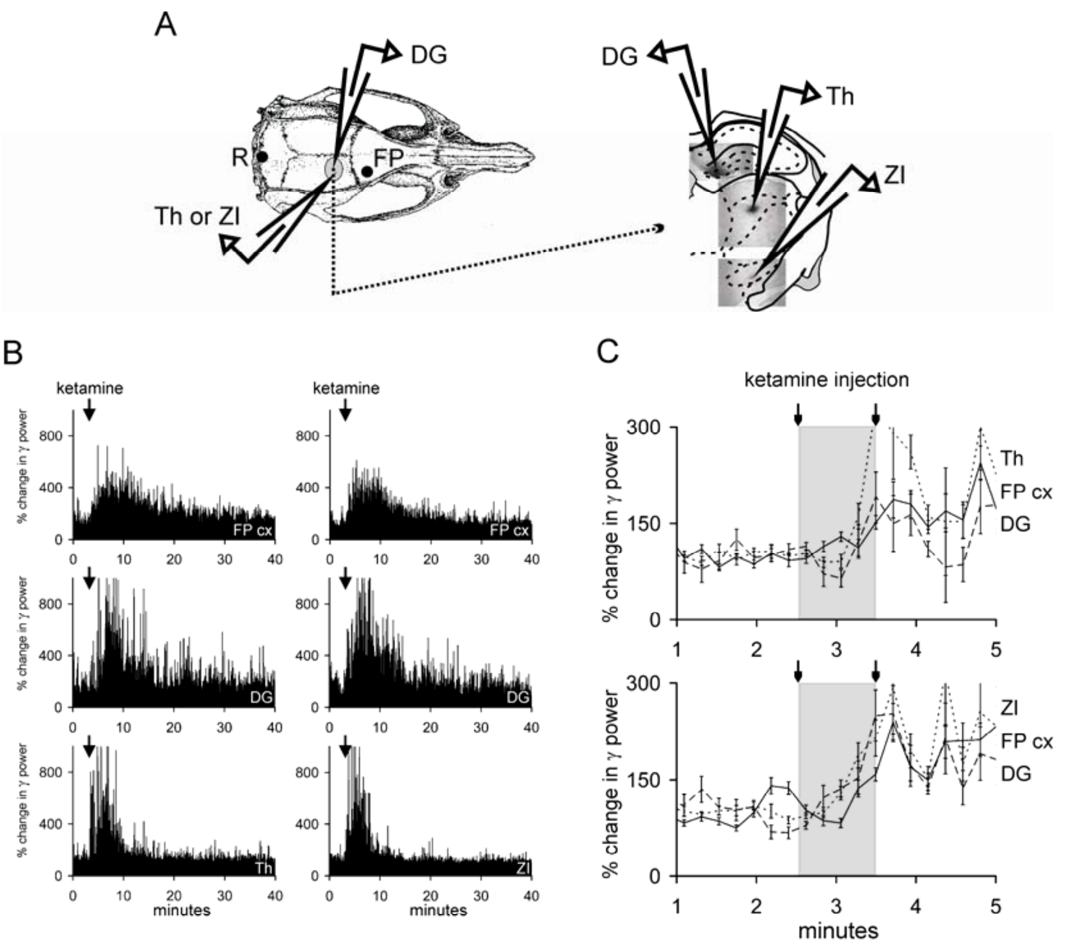

Supplement: Figure S3 — A single intravenous injection of ketamine (0.5 mg/kg) quickly increases the power of γ oscillations in the frontoparietal cortex, hippocampus, thalamus and zona incerta. (A): Experimental design (dorsal and coronal views): In these experiments (N = 3), the frontoparietal (FP) ECoG (or FP cx) and the hippocampal (dentate gyrus or DG) LFP recordings were permanent. On the other hand, a Neurobiotin-ACSF-filled micropipette (tip diameter: 3–7 µm) was moved down in subcortical structures, including the thalamus (Th) and zona incerta (ZI). At the end of the recording session, the neuronal tracer is applied using extracellular iontophoresis (+600 nA, 200 ms on, 200 ms off, for 10 min). The tracer is revealed using a standard ABC-DAB procedure (Pinault, 1996). The microphotographs reveal the location of the recording sites (black spots). (B): The left and right panels show % change in γ power measured from two successive triple recording sessions, FPcx-DG-Th and FPcx-DG-ZI. The second intravenous injection of ketamine was made more than 2 hours after the first injection. Note that ketamine increases the γ power simultaneously at all recording sites. (C): The charts show the quick increase in γ oscillations during the first 2 minutes that followed the onset of intravenous injection of ketamine. The grey areas indicate the period during which ketamine was intravenously injected. Each point is the average of 12–15 successive FFT values (±sem) of γ power. Note that, at all recording sites, the γ power starts to increase during ketamine injection. (0.12 MB PDF) [file pone.0006755.s003.pdf]
